# Resveratrol Confers Vascular Protection by Suppressing TLR4/Syk/NLRP3 Signaling in Oxidized Low-Density Lipoprotein-Activated Platelets

**DOI:** 10.1155/2021/8819231

**Published:** 2021-02-25

**Authors:** Yun Xue, Huilian Chen, Shenghao Zhang, Li Bao, Beidong Chen, Huan Gong, Yanyang Zhao, Ruomei Qi

**Affiliations:** ^1^MOH Key Laboratory of Geriatrics, Beijing Hospital, National Center of Gerontology, Beijing, China; ^2^Graduate School of Peking Union Medical College, Beijing, China

## Abstract

This study investigated the effect of resveratrol on Toll-like receptor 4- (TLR4-) mediated matrix metalloproteinase 3 (MMP3) and MMP9 expression in oxidized low-density lipoprotein- (ox-LDL-) activated platelets and the potential molecule mechanism. Human platelets were used in the present study. The results showed that resveratrol suppressed TLR4, MMP3, and MMP9 expression in ox-LDL-activated platelets. The TLR4 inhibitor CLI-095 also inhibited MMP3 and MMP9 expression and secretion in ox-LDL- and lipopolysaccharide- (LPS-) activated platelets. The combination of resveratrol and CLI-095 synergistically suppressed MMP3 and MMP9 expression in ox-LDL- and LPS-activated platelets. These findings suggest that the resveratrol-induced inhibition of MMP3 and MMP9 expression is linked to the suppression of TLR4 activation. Resveratrol also suppressed spleen tyrosine kinase (Syk) phosphorylation and nucleotide-binding domain leucine-rich repeat containing protein 3 (NLRP3) expression and IL-1*β* secretion in ox-LDL- and LPS-treated platelets. The coimmunoprecipitation results showed that resveratrol inhibited the binding of Syk and NLRP3. Finally, resveratrol reduced vascular senescence cells and the expression of TLR4, MMP3, and MMP9 and prevented alterations of vascular structure in 52-week-old mice. Our findings demonstrated that resveratrol decreased inflammatory protein expression and improved vascular structure in aged mice. Resveratrol inhibited the expression of TLR4 and secretion of MMP3, MMP9, and IL-1*β*. The mechanism of action of resveratrol appears to be associated with the inhibition of TLR4/Syk/NLRP3 activation in ox-LDL-activated platelets.

## 1. Introduction

Cardiovascular diseases are the leading cause of death worldwide. Atherosclerosis is the main pathological characteristic of cardiovascular disease. Atherosclerosis is a chronic vascular inflammatory disease of the arteries, the incidence of which is related to aging [1, 2] and several diseases, such as hypertension [3], hyperlipidemia [4], diabetes [5, 6], and obesity [7]. Harmful metabolite accumulation, such as oxidative products, excessive glycation products, and lipid components, is a main cause of vascular inflammation. Oxidized low-density lipoprotein (ox-LDL) is a main risk factor for atherosclerosis [8]. Oxidized LDL can stimulate platelet activation and release various inflammatory proteins. Platelets are inflammatory cells that have received research attention with regard to the development of atherosclerosis. Platelets are derived from megakaryocytes and contain many *α*-granules and dense granules that store abundant inflammatory mediators. Platelets are involved in the inflammatory process in atherosclerosis by releasing these inflammatory mediators into the blood and recruiting monocytes to damaged vascular endothelial cells. Therefore, platelets appear to play a critical role in the early stages of atherosclerosis.

Toll-like receptors (TLRs) are innate immune receptors that have been widely studied. They recognize exogenous stimuli and also respond to endogenous harmful metabolites [9, 10]. Accumulating evidence indicates that TLR2, TLR3, and TLR4 are involved in atherosclerosis [11, 12]. We recently reported that TLR4 was activated in ox-LDL-stimulated platelets, but the mechanism of TLR4 activation in platelets is not fully understood.

Nucleotide-binding domain leucine-rich repeat containing protein 3 (NLRP3) is an important component of the inflammasome that plays a critical role in pyroptosis [13, 14]. The NLRP3 inflammasome is composed of NLRP3, caspase-1, and the adapter protein apoptosis-associated speck-like protein (ASC) [15]. Upon NLRP3 activation, procaspase-1 is converted to active caspase-1 to promote the maturation of interleukin-1*β* (IL-1*β*) [16]. However, the role of the NLRP3 inflammasome in platelets is still unknown. Previous studies indicated that formation of the NLRP3 inflammasome is promoted by spleen tyrosine kinase (Syk) activation [17]. Syk is a pivotal tyrosine kinase upon platelet activation that is involved in platelet activation that is induced by various agonists, including thrombin [18], collagen [19], and FcR*γ*II [20]. However, the interaction between NLRP3 and Syk in ox-LDL-activated platelets remains to be elucidated.

Matrix metalloproteinases (MMPs) are extracellular zinc proteases. Human MMPs comprise 24 members that can cause focal destruction of the vascular extracellular matrix (ECM) through proteolysis [21]. The components of the ECM include various types of collagens and elastin, fibronectin, and laminin. These components are important for maintaining vascular homeostasis. An increase in MMP levels promotes ECM degradation in the vascular wall, leading to vascular structure disorders and unstable plaque formation. Moreover, an increase in MMPs may facilitate endothelial dysfunction, inflammation, and thrombosis in atherosclerosis [22–24]. Most MMPs are produced by endothelial cells, smooth muscle cells, and inflammatory cells, but the role of MMPs in platelets is less well understood.

Resveratrol is a natural polyphenol that has several pharmacological properties, such as anti-inflammation [25], antioxidation [26], and antiplatelet aggregation [27]. Some studies have shown that resveratrol protects against atherosclerosis and aging [28, 29]. The antiaging effects of resveratrol are associated with an increase in expression of the longevity molecule sirtuin 1 (Sirt1). We recently reported that resveratrol inhibited TLR4 expression and platelet aggregation and increased Sirt1 expression and AMPK phosphorylation in activated platelets [30]. In the present study, we further investigated the effect of resveratrol on MMPs and whether its mechanism of action is linked to the NLRP3/Syk signaling pathway in activated platelets. We also evaluated the protective action of resveratrol on vascular structure in aged mice.

## 2. Materials and Methods

### 2.1. Ethics Statement

Blood was collected from healthy donors. The participants provided written informed consent to provide blood samples. The experiments were conducted according to the principles of the Declaration of Helsinki (World Medical Association, 2013). The blood samples were used for the *in vitro* study. The present study was approved by the Ethics Committee of the Beijing Institute of Geriatrics (no. 201506).

### 2.2. Materials

Resveratrol (R5010, >99% purity) was purchased from Sigma-Aldrich (St. Louis, MO, USA). CLI-095 (synonymous with TAK-242), R788, and MCC950 were obtained from MedChemExpress (Monmouth Junction, NJ, USA). Antibodies against MMP3 (catalog no. ab52915), MMP9 (catalog no. ab38898), caspase-1 (catalog no. ab1872), IL-1*β* (catalog no. ab226918), and TLR4 (catalog no. ab8378) were purchased from Abcam (Boston, MA, USA). The antibody against NLRP3 (catalog no. 13158) was obtained from Cell Signaling Technology (Beverly, MA, USA). Antibodies against *β*-actin (catalog no. sc-47778), TLR4 (catalog no. sc-293072), p21 (catalog no. sc-56335), phosphorylated p53 (p-p53; catalog no. sc-18079), p53 (catalog no. sc-263), and Syk (catalog no. sc-73086) were purchased from Santa Cruz Biotechnology (Santa Cruz, CA, USA). The antibody against tyrosine phosphorylated 4G10 (catalog no. 05-321) was obtained from Millipore (Billerica, MA, USA). Human MMP3 and MMP9 and mouse MMP9 enzyme-linked immunosorbent assay (ELISA) kits were obtained from R&D Systems (Minneapolis, MN, USA). The mouse MMP3 and mouse IL-1*β* ELISA kits were obtained from Abcam (Boston, MA, USA).

### 2.3. Preparation of Platelets

Venous blood was collected from healthy volunteers who did not take any medications using 3.8% sodium citrate as an anticoagulant. Platelet-rich plasma (PRP) was obtained from whole blood by centrifugation at 200 × *g* for 10 min. The PRP was then centrifuged at 1400 rotations per minute (rpm) for 10 min in the presence of ACD and 2 mM ethylenediaminetetraacetic acid (EDTA), washed twice with modified Tyrode's buffer (138 mM NaCl, 3.3 mM NaH_2_PO_4_·2H_2_O, 1 mM MgCl_2_, 2.9 mM KCl, 5.5 mM glucose, and 20 mM HEPES), and resuspended in modified Tyrode's buffer.

### 2.4. Preparation of LDL and ox-LDL

Human LDL was isolated from fresh serum by sequential ultracentrifugation. LDL was oxidized with CuSO4 (5 *μ*M) for 16 h at 37°C, and then, the oxidation was stopped by the addition of EDTA (20 *μ*M). Protein concentration of ox-LDL was determined by using Thermo Scientific Multiskan FC microplate reader (Waltham, Massachusetts, USA). The ox-LDL preparation was filtered through 0.22 *μ*m filters and stored at 4°C.

### 2.5. Flow Cytometry Analysis of TLR4 Expression

The platelet suspension (1 × 10^6^/ml) was treated with or without resveratrol (100 *μ*M) for 10 min. Afterward, ox-LDL (0.1 mg/ml) was added for another 10 min at 37°C without stirring. The platelets were fixed by the addition of 4% paraformaldehyde for 10 min. After washing three times, the platelets were incubated with PE-conjugated CD61 and FITC-conjugated TLR4 for 30 min. The platelets were analyzed on a FACScan flow cytometer (BD Bioscience) with 10000 events per gate and analyzed using FlowJo software.

### 2.6. Platelet Spreading

Glass slides were coated with 20 *μ*g/ml fibrinogen overnight, and then, a 2 × 10^6^ platelet suspension (100 *μ*l) was added on the glass slides for 1 h at room temperature. Nonadherent platelets were removed by aspiration, washed twice with phosphate-buffered saline (PBS), fixed with 4% paraformaldehyde, permeabilized by the addition of 0.1% TritonX-100, and stained with Phalloidin-iFluor 555 (Abcam, Boston, MA, USA) for 1 h. Platelet spreading was visualized by confocal fluorescence microscopy (Nikon Instruments, Melville, NY, USA).

### 2.7. Immunofluorescent Staining

The platelets were incubated with resveratrol (100 *μ*M) for 10 min, and then, ox-LDL (0.1 mg/ml) was added for another 10 min. The platelets were fixed with 1% paraformaldehyde, adhered on 0.01% poly-L-lysine-coated coverslips, and permeabilized with 0.1% TritonX-100. The platelets were stained with anti-caspase-1 (1 : 100) overnight at 4°C and then washed with PBS three times and incubated with DyLight 549 AffiniPure goat anti-rabbit IgG (1 : 200, EarthOx, San Francisco, CA, USA) for 1 h at room temperature. Images were captured using a confocal fluorescence microscope (Nikon Instruments, Tokyo, Japan).

### 2.8. Immunoblotting and Immunoprecipitation

For the Western blot assay, platelets (1 × 10^9^/ml) were preincubated with resveratrol (1, 10, and 100 *μ*M), CLI-095 (0.1, 0.3, and 1 *μ*M), R788 (10, 30, and 100 nM), or MCC950 (1, 10, and 100 nM) for 10 min. Proteins were separated by sodium dodecyl sulfate-polyacrylamide gel electrophoresis (PAGE) and transferred to polyvinylidene membranes. The membranes were incubated overnight with primary antibodies (1 : 1000) at 4°C and then incubated with anti-mouse or anti-rabbit antibodies (1 : 5000). The bands were exposed using an ECL chemiluminescent reagent and the EvolutionCapt system (Vilber Lourmat) and quantified using ImagePro Plus software.

For immunoprecipitation, platelets were treated with resveratrol (100 *μ*M) for 10 min, and then, ox-LDL (0.1 mg/ml) was added for 10 min. Platelets were lysed by the addition of RIPA buffer (100 mM Tris-HCl (pH 7.5), 2% Tritox-100, 2 mM EGTA, 200 mM NaCl, 2 *μ*M phenylmethylsulfonyl fluoride (PMSF), and 10 *μ*g/ml leupeptin). An antibody against p-Syk or NLRP3 and normal rabbit IgG were used for protein precipitation. Lysates were incubated with 1 *μ*g of primary antibodies (monoclonal anti-phosphorylate Syk antibody or monoclonal anti-NLRP3 antibody) at 4°C for 1 h, and then, protein A/G beads (100 *μ*l) were added overnight at 4°C. Immunoblot analysis was used for protein expression.

### 2.9. Measurement of MMP3, MMP9, and IL-1*β* by ELISA

Platelets were treated with or without resveratrol (100 *μ*M) for 10 min, and then, ox-LDL (0.1 mg/ml) was added for 10 min. After centrifugation at 10000 rpm for 10 min, the supernatant was collected to measure MMP3, MMP9, and IL-1*β* using ELISA kits according to the manufacturer's instructions.

### 2.10. Animal Experiments

Male C57/BL mice (8 and 52 weeks old) were purchased from Vital River Laboratory Animal Technology Co., Ltd. (Beijing, China). All of the animal experiments were performed in accordance with guidelines for animal experimentation of the National Institutes of Health and were approved by the Ethics Committee of the National Center of Gerontology. The mice were housed in cages with constant humidity and temperature, free access to food and water, and a 12 h/12 h light/dark cycle. The mice were given normal mouse chow or high-fat chow that contained 21% fat supplemented with 1.25% cholesterol. The mice (8 and 52 weeks old) were randomly divided into three groups (*n* = 10/group): normal chow group (control), high-fat chow group (high-fat), and high-fat chow treated with resveratrol (high-fat+RSV). The control group and high-fat chow group were given 0.3 ml of physiological saline per day as the vehicle for 12 weeks. Resveratrol (22.4 mg/kg/day) was administered intragastrically in the high-fat chow+RSV group for 12 weeks [31]. Body weight was recorded every 2 weeks. At the end of 12 weeks of treatment, the mice were intraperitoneally anesthetized with sodium pentobarbital. Blood samples and aortas were harvested for further studies.

### 2.11. Sirius Red Staining

To observe collagen components, we used Sirius red staining. The Sirius staining kit was purchased from Solarbio Science and Technology Co. (Beijing, China). The sections were stained with Weigert iron Sumu essence dye for 15 min, rinsed for 5 min, and then washed with distilled water. The sections were covered with 200 *μ*l Sirius red dye for 1 h. The scanned slides were viewed at 200x magnification using an Olympus microscope with a Nikon CCD system (Tokyo, Japan).

### 2.12. Senescence *β*-Galactosidase Staining

Aortic root sections were stained using a senescence *β*-galactosidase (SA-*β*-gal) commercial kit (Beyotime, Beijing, China) to assess SA-*β*-gal activity according to the manufacturer's protocol. Briefly, the sections were fixed in fixative solution at room temperature for 15 min and washed with PBS three times. The sections were then incubated overnight at 37°C in staining solution. Blue cells were SA-*β*-gal-positive, evaluated by using a light microscope (Nikon, Tokyo, Japan).

### 2.13. Immunohistochemistry

Aortic root specimens were dissected under a stereomicroscope, fixed in a 4% formaldehyde solution, and frozen in an optimal-cutting-temperature embedding medium for serial cryosectioning of the aortic root specimens. Immunohistochemical staining with specific antibodies was assessed using multiple samples of each artery. A bound antibody was detected with a DAB substrate kit (Beijing Zhongshan Golden Bridge Biotechnology, Beijing, China). Semiquantitative immunostaining analysis was performed using an Olympus microscope that was linked to the ImagePro Plus image analysis system (Media Cybernetics, Bethesda, MD, USA).

### 2.14. Statistical Analysis

Quantitative data are expressed as mean ± SEM. Significant differences between two groups were analyzed using two-tailed unpaired Student's *t*-test. Statistical significance among multiple groups was analyzed using one-way analysis of variance (ANOVA) followed by the Student-Newman-Keuls *post hoc* test. All of the analyses were performed using SPSS 21.0 software (Armonk, NY, USA). Values of *p* < 0.05 were considered statistically significant.

## 3. Results

### 3.1. Resveratrol Inhibited Platelet Spreading, TLR4 Expression, and Secretion of MMP3 and MMP9 in ox-LDL-Activated Platelets

**Figure 1:**
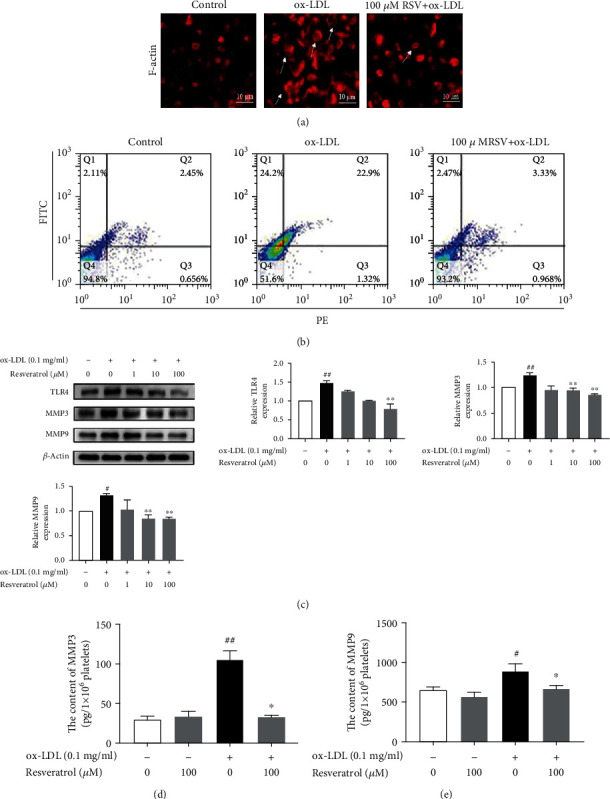
Resveratrol inhibited platelet spreading and TLR4, MMP3, and MMP9 expression in ox-LDL-activated platelets. (a, b) Platelets were treated with or without resveratrol (100 *μ*M) for 10 min, and then, ox-LDL (0.1 mg/ml) was added for another 10 min. (a) Immunofluorescence analysis of platelet spreading. (b) Flow cytometry analysis of TLR4 expression on the platelet membrane. (c) Protein levels of TLR4, MMP3, and MMP9 were analyzed by Western blot. (d) MMP3 secretion in platelets was measured by using an ELISA kit. (e) MMP9 secretion in platelets was measured by using an ELISA kit. The data represent three independent experiments. ^#^*p* < 0.05, ^##^*p* < 0.01, significant difference between non-ox-LDL-treated platelets and ox-LDL-treated platelets; ^∗^*p* < 0.05, ^∗∗^*p* < 0.01, significant difference between resveratrol-treated platelets and ox-LDL-treated platelets alone.

Platelet adhesion and spreading are important pathological phenomena in atherosclerosis. Our previous study found that 100 *μ*M resveratrol inhibited platelet aggregation that was induced by thrombin receptor-activated peptide and collagen. In the present study, we used the same concentration of resveratrol to detect its effect on platelet spreading. As shown in Figure 1(a), ox-LDL treatment caused platelets to have a pseudopod appearance and spread. Less spreading was observed in resveratrol-treated platelets. We recently reported that TLR4 was involved in ox-LDL-activated platelets. In the present study, we also investigated whether TLR4 activation is associated with MMP secretion. TLR4 expression was analyzed by flow cytometry and Western blot. The flow cytometry results showed that ox-LDL treatment increased membrane TLR4 expression by 20.6% (Figure 1(b)). The Western blot results showed that TLR4 expression increased 2.8-fold in ox-LDL-treated platelets, and resveratrol treatment inhibited this TLR4 expression. Matrix metalloproteinases are involved in atherosclerosis by degrading collagen content and inflammation, but the role of MMPs in platelets is still unclear. Therefore, we evaluated MMP3 and MMP9 expression in ox-LDL-treated platelets. As shown in Figure 1(c), MMP3 and MMP9 expression increased 2.31- and 2.67-fold in ox-LDL-treated platelets, respectively. Resveratrol dose-dependently inhibited MMP3 and MMP9 expression that was induced by ox-LDL. We also measured MMP3 and MMP9 secretion. As shown in Figures 1(d) and 1(e), resveratrol treatment reduced MMP3 and MMP9 secretion that was induced by ox-LDL. The content of MMP3 was 30.2 ± 8.2 pg/ml in non-ox-LDL-treated platelets and 104.7 ± 23.7 pg/ml in ox-LDL-treated platelets. Resveratrol significantly reduced MMP3 levels to 32.4 ± 5.7 pg/ml. We also measured MMP9 levels, which were 651.7 ± 71.9 pg/ml in non-ox-LDL-treated platelets and 887.7 ± 193.4 pg/ml in ox-LDL-treated platelets. Resveratrol treatment significantly decreased MMP9 levels to 670.2 ± 76.8 pg/ml.

### 3.2. Inhibition of MMP3 and MMP9 Expression by Resveratrol Was Related to the Blockade of TLR4 Signaling

**Figure 2:**
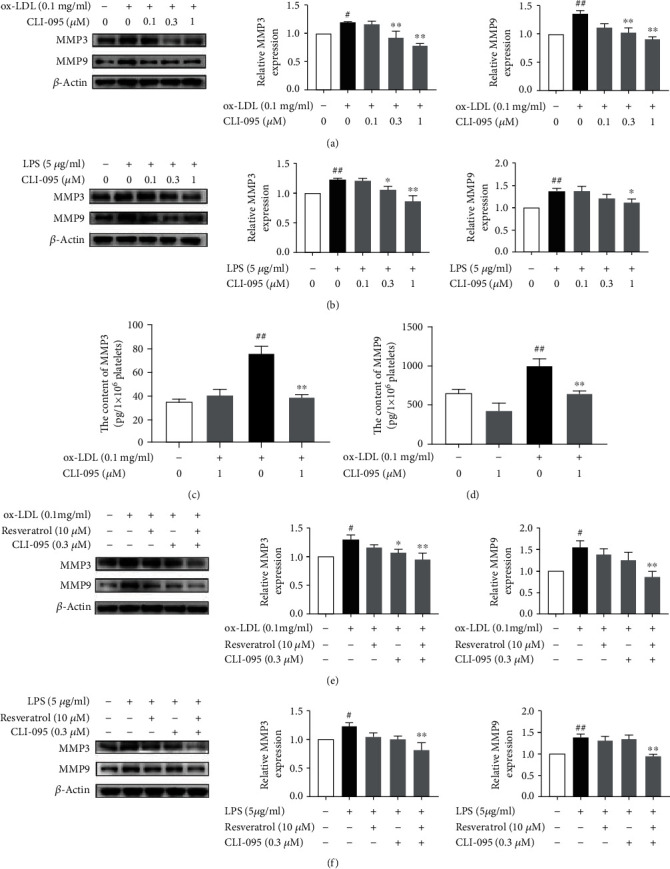
Treatment with the combination of CLI-095 and resveratrol synergistically inhibits MMP3 and MMP9 expression in ox-LDL- and LPS-activated platelets. Platelets were pretreated with or without CLI-095 (0.1, 0.3, and 1 *μ*M) for 10 min, and then, ox-LDL (0.1 mg/ml) was added for another 10 min. (a) CLI-095 inhibited MMP3 and MMP9 expression that was induced by ox-LDL. (b) CLI-095 inhibited MMP3 and MMP9 expression that was induced by LPS. (c) Resveratrol inhibited MMP3 secretion that was induced by ox-LDL. (d) Resveratrol inhibited MMP9 secretion that was induced by ox-LDL. (e) Resveratrol and CLI-095 synergistically inhibited MMP3 and MMP9 expression that was induced by ox-LDL. (f) Resveratrol and CLI-095 synergistically inhibited MMP3 and MMP9 expression that was induced by LPS. The data represent three independent experiments. ^#^*p* < 0.05, ^##^*p* < 0.01, significant difference between non-ox-LDL-treated platelets and ox-LDL- or LPS-treated platelets; ^∗^*p* < 0.05, ^∗∗^*p* < 0.01, significant difference between CLI-095-treated platelets and ox-LDL- or LPS-activated platelets.

Next, we investigated whether the inhibitory effect of resveratrol on MMP3 and MMP9 expression occurs through the suppression of TLR4 signaling. The TLR4-specific agonist LPS and TLR4-specific signaling inhibitor CLI-095 were used. As shown in Figures 2(a) and 2(b), MMP3 and MMP9 expression was significantly increased by ox-LDL (0.1 mg/ml) and LPS (5 *μ*g/ml). CLI-095 (1 *μ*M) significantly suppressed the expression of MMP3 and MMP9 that was induced by ox-LDL and LPS. The secretion of MMP3 and MMP9 was then detected. As shown in Figures 2(c) and 2(d), CLI-095 decreased MMP3 content that was induced by ox-LDL (38.44 ± 3.76 pg/ml in the CLI-095-treated group *vs.*71.05 ± 4.34 pg/ml in the ox-LDL-treated group). CLI-095 also inhibited MMP9 secretion that was induced by ox-LDL. The level of MMP9 was 645.12 ± 52.48 pg/ml in the CLI-095-treated group *vs.*999.03 ± 145.85 pg/ml in the ox-LDL-treated group. A significant difference was found between the CLI-095 group and ox-LDL group.

To confirm that the inhibitory effect of resveratrol on MMP3 and MMP9 expression is associated with the inhibition of TLR4 activation, we evaluated whether treatment with the combination of resveratrol and CLI-095 synergistically inhibits MMP3 and MMP9 expression in ox-LDL- and LPS-activated platelets. Lower doses of CLI-095 (0.3 *μ*M) and resveratrol (10 *μ*M) were tested. As shown in Figures 2(e) and 2(f), treatment with CLI-095 (0.3 *μ*M) or resveratrol (10 *μ*M) alone only partially suppressed MMP3 and MMP9 expression. The combination of CLI-095 and resveratrol completely abolished MMP3 and MMP9 expression in ox-LDL- and LPS-activated platelets.

### 3.3. Resveratrol Inhibited Syk Phosphorylation in ox-LDL-Treated Platelets

**Figure 3:**
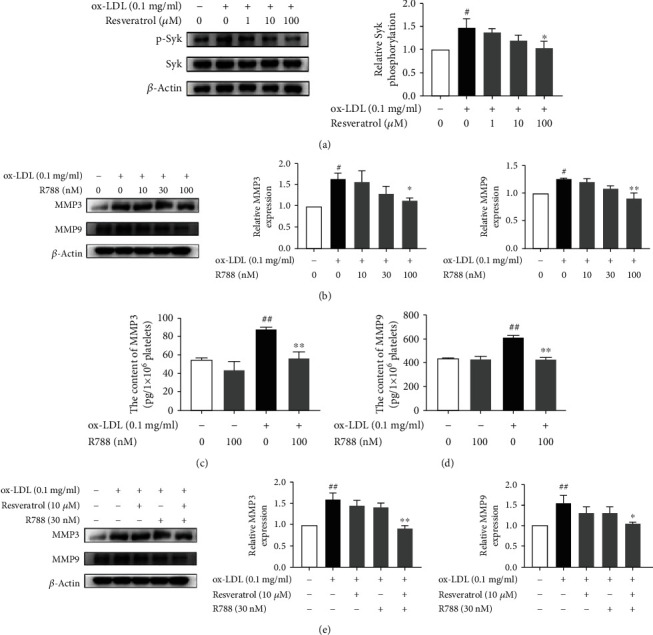
Resveratrol inhibited Syk phosphorylation in ox-LDL-treated platelets. (a) Resveratrol dose-dependently inhibited Syk phosphorylation that was induced by ox-LDL. (b) The Syk inhibitor R788 inhibited MMP3 and MMP9 expression that was induced by ox-LDL. (c) R788 (100 nM) decreased MMP3 secretion that was induced by ox-LDL. (d) R788 (100 nM) reduced MMP9 secretion that was induced by ox-LDL. (e) R788 (30 nM) and resveratrol (10 *μ*M) synergistically inhibited MMP3 and MMP9 expression that was induced by ox-LDL. The data represent three independent experiments. ^#^*p* < 0.05, ^##^*p* < 0.01, significant difference between non-ox-LDL-treated platelets and ox-LDL-treated platelets; ^∗^*p* < 0.05, ^∗∗^*p* < 0.01, significant difference between R788-treated platelets and ox-LDL-activated platelets.

Syk is an important signaling molecule in various types of cell [18]. Previous studies showed that Syk activation is associated with TLR4 signaling [32]. To identify whether the effect of resveratrol on MMP3 and MMP9 expression is associated with Syk activation, we detected Syk phosphorylation in the present study. As shown in Figures 3(a)–3(d), ox-LDL stimulation elevated Syk phosphorylation 3.18-fold. Resveratrol dose-dependently suppressed the phosphorylation of Syk. To evaluate whether Syk inhibition influences MMP3 and MMP9 expression, we used the Syk inhibitor R788. R788 (10, 30, and 100 nM) dose-dependently attenuated MMP3 and MMP9 expression. R788 at a dose of 100 nM completely blocked MMP3 and MMP9 expression and secretion that were induced by ox-LDL. We also investigated whether the combination of R788 and resveratrol synergistically inhibits MMP3 and MMP9. As shown in Figure 3(e), resveratrol (10 *μ*M) or R788 (30 nM) treatment alone did not completely inhibit MMP3 or MMP9 expression that was induced by ox-LDL, whereas the combination of resveratrol and R788 completely abolished MMP3 and MMP9 expression. Similar data were found in LPS-treated platelets (Supplementary Figure S1).

### 3.4. Resveratrol Inhibited NLRP3/Caspase-1/IL-1*β* Expression and Secretion in ox-LDL-Treated Platelets

**Figure 4:**
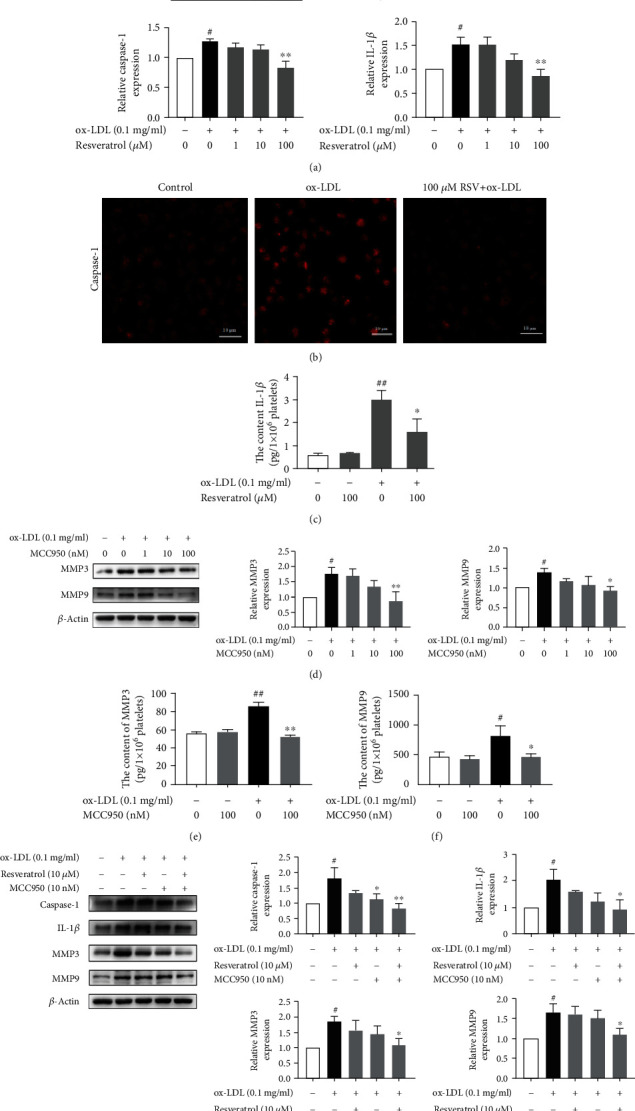
Resveratrol suppressed NLRP3/caspase-1/IL-1*β* expression and secretion in ox-LDL-treated platelets. (a) Resveratrol inhibited NLRP3, caspase-1, and IL-1*β* expression that was induced by ox-LDL. (b) Immunofluorescent analysis of caspase-1 expression. Resveratrol (100 *μ*M) decreased caspase-1 expression. (c) Resveratrol (100 *μ*M) reversed the increase in IL-1*β* secretion in ox-LDL-activated platelets. (d) The NLRP3 inhibitor MCC950 inhibited MMP3 and MMP9 expression that was induced by ox-LDL. (e) MCC950 (100 nM) decreased MMP3 secretion in ox-LDL-activated platelets. (f) MCC950 (100 nM) decreased MMP9 secretion in ox-LDL-activated platelets. (g) The combination of resveratrol (10 *μ*M) and MCC950 (10 nM) synergistically inhibited the expression of caspase-1, IL-1*β*, MMP3, and MMP9 that was induced by ox-LDL. The data represent three independent experiments. ^#^*p* < 0.05, ^##^*p* < 0.01, significant difference between non-ox-LDL-treated platelets and ox-LDL-treated platelets; ^∗^*p* < 0.05, ^∗∗^*p* < 0.01, significant difference between MCC950- or MCC950+resveratrol-treated platelets and ox-LDL-activated platelets.

The NLRP3 inflammasome plays an important role in the inflammatory response. However, the role of the NLRP3 inflammasome in platelets is less well understood. NLRP3 activity, reflected by caspase-1 activation and cleavage and IL-1*β* secretion, was evaluated in the present study. As shown in Figures 4(a)–4(c), ox-LDL stimulation significantly enhanced NLRP3, caspase-1, and IL-1*β* expression, and 100 *μ*M resveratrol completely suppressed their expression. Resveratrol (100 *μ*M) treatment reduced caspase-1 immunofluorescence intensity compared with ox-LDL-treated platelets. Moreover, IL-1*β* secretion was determined using an ELISA kit. Resveratrol (100 *μ*M) significantly decreased IL-1*β* secretion. The level of IL-1*β* was 3.02 ± 0.67 pg/ml in the ox-LDL-treated group and 1.42 ± 0.69 pg/ml in the resveratrol-treated group. A significant difference was found between the resveratrol-treated group and ox-LDL-treated group. We then tested the effect of the specific NLRP3 inhibitor MCC950 on caspase-1, IL-1*β*, MMP3, and MMP9 expression. As shown in Figures 4(d)–4(f), MCC950 (100 nM) significantly suppressed MMP3 and MMP9 expression that was induced by ox-LDL. MCC950 also decreased the secretion of MMP3 and MMP9 in ox-LDL-treated platelets. The levels of MMP3 were 52.72 ± 2.19 pg/ml in the MCC950-treated group and 86.84 ± 5.79 pg/ml in the ox-LDL-treated group. The levels of MMP9 were 467.52 ± 84.26 pg/ml in the MCC950-treated group and 827.93 ± 335.71 pg/ml in the ox-LDL-treated group, which were significantly different. We also evaluated possible synergistic effects of the combination of resveratrol and MCC950 on the expression of caspase-1, IL-1*β*, MMP3, and MMP9. As shown in Figure 4(g), resveratrol (10 *μ*M) and MCC950 (10 nM) treatment alone only partially inhibited the expression of caspase-1, IL-1*β*, MMP3, and MMP9, whereas treatment with the combination of resveratrol and MCC950 abolished the expression of caspase-1, IL-1*β*, MMP3, and MMP9 in ox-LDL-activated platelets. A similar inhibitory effect of MCC950 on caspase-1 and IL-1*β* expression and secretion was observed in LPS-activated platelets (Supplementary Figure S2).

### 3.5. Resveratrol Reversed the ox-LDL-Induced Binding of Syk/NLRP3 and TLR4 in Activated Platelets

**Figure 5:**
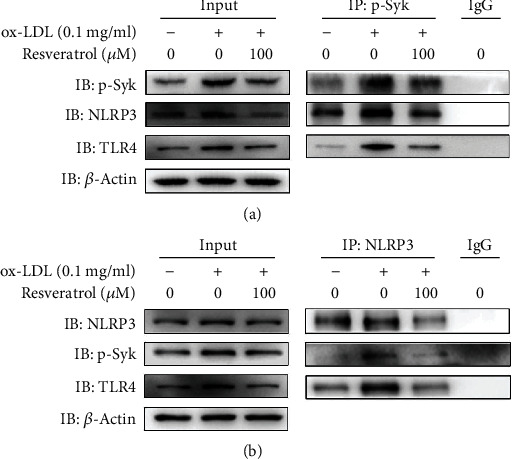
Resveratrol reversed the ox-LDL-induced binding of Syk/NLRP3 and TLR4 in activated platelets. The binding of Syk/NLRP3 and TLR4 was analyzed by coimmunoprecipitation. Rabbit IgG was used as a nonimmune control. Input represents whole platelet lysate. (a) Anti-phosphorylated Syk antibody precipitated phosphorylated Syk/NLRP3 and TLR4 in ox-LDL-activated platelets, which was reversed by resveratrol. (b) Anti-NLRP3 antibody precipitated phosphorylated Syk/NLRP3 and TLR4 in ox-LDL-activated platelets, which was reversed by resveratrol. The data represent three independent experiments.

Based on the above results, we investigated whether binding between phosphorylated Syk and NLRP3 occurs in ox-LDL-activated platelets. We performed coimmunoprecipitation using an anti-phosphorylated Syk antibody and an anti-NLRP3 antibody. As shown in Figures 5(a) and 5(b), an anti-phosphorylated Syk antibody coimmunoprecipitated phosphorylated Syk and NLRP3 and an anti-NLRP3 antibody immunoprecipitated NLRP3 and phosphorylated Syk in ox-LDL-activated platelets. The results also indicated that regardless of whether Syk or NLRP3 immunoprecipitation occurred, TLR4 was coimmunoprecipitated, suggesting that TLR4, Syk, and NLRP3 together form a loop of inflammation, and resveratrol inhibits TLR4/Syk/NLRP3 signaling.

### 3.6. Resveratrol Decreased p21 and p53 Expression in ox-LDL-Activated Platelets

**Figure 6:**
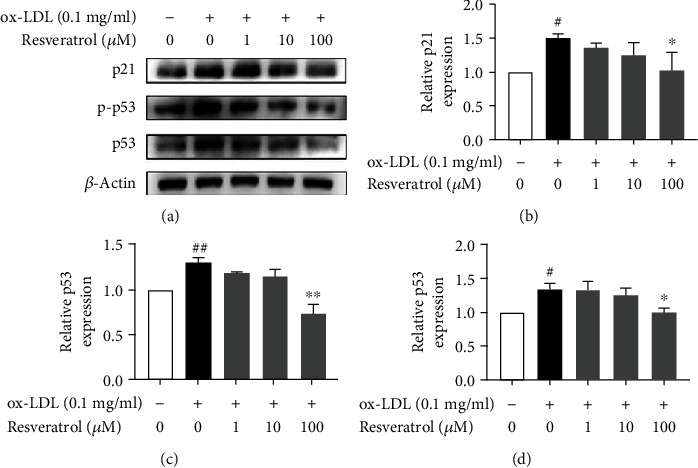
Resveratrol decreased p21 and p53 expression in ox-LDL-activated platelets. (a) Resveratrol decreased p21 expression in ox-LDL-activated platelets. (b) Density analysis of p21 expression. (c) Resveratrol decreased p53 expression in ox-LDL-activated platelets. (d) Density analysis of p53 expression. The data represent three independent experiments. ^#^*p* < 0.05, significant difference between non-ox-LDL-treated platelets and ox-LDL-treated platelets; ^∗^*p* < 0.05, ^∗∗^*p* < 0.01, significant difference between resveratrol-treated platelets and ox-LDL-activated platelets.

p21 and p53 are aging-associated proteins [33, 34]. p53 plays an important role in cell-intrinsic responses to genome instability, including transient cell cycle arrest, senescence, and apoptosis [35]. However, whether resveratrol influences p21 and p53 in ox-LDL-activated platelets remains unclear. To explore this issue, we investigated the effect of resveratrol on the expression of p21 and p53 in platelets. As shown in Figures 6(a)–6(d), ox-LDL increased p21 expression 2.34-fold and resveratrol dose-dependently suppressed this phenotypic change. We then detected changes in p53 expression. Oxidized LDL stimulation enhanced the expression and phosphorylation of p53 1.86-fold in platelets. Resveratrol (100 *μ*M) completely inhibited the increase in p53 expression that was induced by ox-LDL.

### 3.7. Resveratrol Reduced Vascular Senescence Cells and the Expression of TLR4, MMP3, and MMP9 in Mice

**Figure 7:**
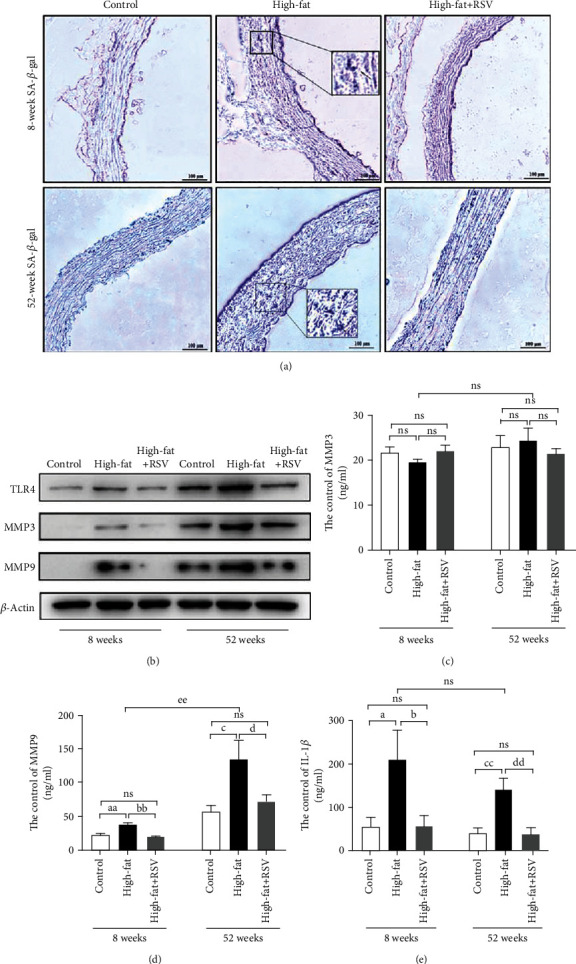
Resveratrol reduced vascular senescence cells and the expression of TLR4, MMP3, and MMP9 in mice. Resveratrol was dispersed in 0.9% NaCl solution and administered intragastrically at a dose of 22.4 mg/kg/day for 12 weeks. The mice were divided into a normal diet group, high-fat diet group, and high-fat diet plus resveratrol group (*n* = 10/group). (a) Senescence cells were detected by SA-*β*-gal staining. Blue cells represent senescence cells. (b) TLR4, MMP3, and MMP9 protein expression in aortic root specimens in each group. (c) Analysis of plasma MMP3 levels in each group. (d) Analysis of plasma MMP9 levels in each group. (e) Analysis of plasma IL-1*β* level in each group. NS: nonsignificant difference between groups. ^aa^*p* < 0.01, significant difference between the 8-week-old control group and high-fat diet group; ^bb^*p* < 0.01, significant difference between the 8-week-old control group and high-fat diet group; ^c^*p* < 0.01, significant difference between the 52-week-old control group and high-fat diet group; ^d^*p* < 0.01, significant difference between the 52-week-old high-fat diet group and resveratrol group.

Age is positively associated with vascular dysfunction in atherosclerosis. We investigated the effect of resveratrol on TLR4, MMP3, and MMP9 in aortic root specimens in 8- and 52-week-old mice. Eight- and 52-week-old mice were given high-fat chow alone or high-fat chow with resveratrol for 12 weeks. We first observed senescence cells in aortic root specimens in 8- and 52-week-old mice that were fed the high-fat diet using SA-*β*-gal staining. As shown in Figure 7(a), compared with 8-week-old mice, the number of senescence cells significantly increased in 52-week-old mice. In contrast, resveratrol-treated 8- or 52-week-old mice exhibited a decrease in senescence cells. Moreover, vascular TLR4, MMP3, and MMP9 expression was analyzed by Western blot. As shown in Figure 7(b), the levels of TLR4, MMP3, and MMP9 expression were higher in high-fat-fed 52-week-old mice compared with high-fat-fed 8-week-old mice. Resveratrol significantly decreased TLR4, MMP3, and MMP9 expression in both groups. Plasma MMP9 expression increased 2.34-fold in high-fat-fed 52-week-old mice. MMP9 levels increased 1.55-fold in high-fat-fed 8-week-old mice, and resveratrol significantly decreased MMP9 levels in high-fat-fed 52-week-old mice. However, plasma MMP3 levels were not different between 8- and 52-week-old mice (Figures 7(c) and 7(d)). Plasma IL-1*β* levels increased 3.75-fold in high-fat-fed 8-week-old mice and increased 3.36-fold in high-fat-fed 52-week-old mice. Resveratrol treatment significantly decreased IL-1*β* levels in both groups (Figure 7(e)).

### 3.8. Resveratrol Inhibited Vascular MMP3 and MMP9 Expression in Aged Mice

**Figure 8:**
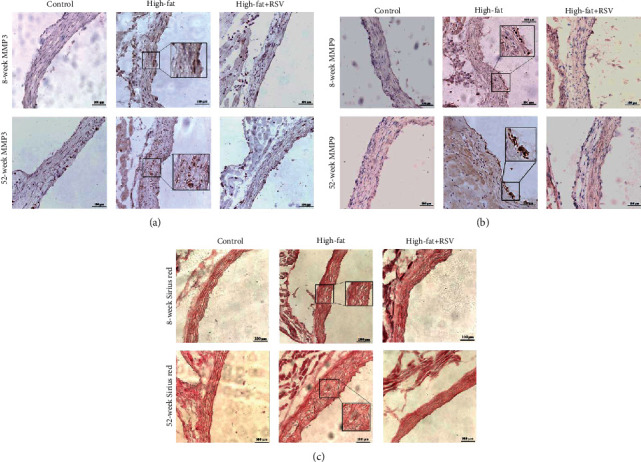
Resveratrol inhibited vascular MMP3 and MMP9 expression in aged mice. Eight- and 52-week-old mice were randomly divided into a control group, high-fat diet group, and high-fat diet plus resveratrol group (high-fat+RSV). (a) Immunohistochemical staining of MMP3 in aortic root sections. (b) Immunohistochemical staining of MMP9 in aortic root sections. (c) Sirius red staining of collagen content in each group.

We then analyzed the expression of MMP3 and MMP9 in aortic root specimens. As shown in Figures 8(a) and 8(b), MMP3 and MMP9 expression was higher in 52-week-old mice than in 8-week-old mice. Resveratrol treatment reduced the expression of both MMP3 and MMP9. Notably, the increase in MMP9 expression was more apparent in 52-week-old mice. These results were consistent with the changes in plasma MMP3 and MMP9 expression. We analyzed vascular collagen structure in aortic root specimens in mice using Sirius red staining. As shown in Figure 8(c), the collagen structure appeared disordered and broken in the aorta in high-fat-fed 8- and 52-week-old mice, and these phenotypic changes were more apparent in 52-week-old mice. In contrast, arrangement of the collagen structure was regular in resveratrol-treated 8- and 52-week-old mice.

## 4. Discussion

The present results showed that resveratrol inhibited TLR4-meidated MMP3 and MMP9 expression and secretion. The mechanism of action of resveratrol appeared to be linked to the inhibition of Syk phosphorylation and NLRP3 inflammasome activation in ox-LDL-treated platelets. Our results also showed increases in NLRP3-associated caspase-1 expression and IL-1*β* secretion in ox-LDL-activated platelets. The inhibition of MMP3 and MMP9 expression and secretion was revealed by TLR4, Syk, and NLRP3 inhibitors, respectively. Recent studies also reported that activated platelets have the ability to produce mature IL-1*β* [36–38]. However, the mechanism of this process in platelets has remained elusive.

The tyrosine kinase Syk is a key modulator of platelet activation and immune signaling. Syk is a nonreceptor tyrosine kinase protein that can bind to immune cell receptors of the intracellular signaling pathway and facilitate the initiation of inflammatory responses. Syk was shown to bind to the immunoreceptor tyrosine-based activation motif-associated receptor (FcR*γ*II) and triggering receptor expressed on myeloid cells-1 (TREM-1; i.e., an effector molecule of the innate immune response) [39, 40]. The present results showed that Syk plays a crucial role in TLR4-mediated MMP3 and MMP9 expression and secretion. Both resveratrol and the Syk inhibitor exerted an inhibitory effect on MMP3 and MMP9 expression and secretion in ox-LDL-activated platelets. This suggests an interaction between Syk and TLR4, and Syk is likely an important pharmacological target of resveratrol. Resveratrol inhibited TRL4 and Syk activation in ox-LDL-activated platelets. We also evaluated NLRP3 inflammasome activation in the present study. The inflammasome is associated with many chronic aging-related diseases, such as atherosclerosis and other cardiovascular diseases. Chronic inflammation has been shown to occur in the vascular system with age, the underlying mechanism of which is linked to natural immunity and inflammation, but the effect of resveratrol on this process in atherogenesis has remained unclear. The present study explored the interaction between Syk and NLRP3 in ox-LDL-activated platelets. Coimmunoprecipitation showed that Syk and NLRP3 were simultaneously precipitated in ox-LDL-activated platelets. We also found that resveratrol attenuated NLRP3-associated caspase-1 expression and IL-1*β* secretion in ox-LDL-activated platelets. This suggests that resveratrol inhibits the platelet inflammatory response by inhibiting multiple molecular targets, including the NLRP3 inflammasome. Platelets are rich in expression of the NLRP3 inflammasome [37, 41]. A recent study reported that the platelet NLRP3 inflammasome was upregulated in a murine model of pancreatic cancer and promoted platelet aggregation and tumor growth. Platelets mediate the increase in endothelium permeability in dengue through NLRP3 inflammasome activation [38]. The NLRP3 inflammasome and Bruton's tyrosine kinase in platelets coregulate platelet activation [37]. The present study provides further evidence that ox-LDL and LPS stimulated TLR4/Syk/NLRP3 activation and resveratrol suppressed MMP3 and MMP9 expression and secretion by inhibiting the TLR4/Syk/NLRP3 inflammasome loop in ox-LDL- and LPS-activated platelets.

Aging and ox-LDL are risk factors for vascular aging and atherosclerosis. The expression of p21 and p53 is associated with aging [42, 43]. The present study showed that resveratrol downregulated p21 and p53 expression in ox-LDL-activated platelets. We also found that a high-fat diet promoted vascular cell senescence in 8- and 52-week-old mice, and this effect was more pronounced in 52-week-old mice. TLR4, MMP3, and MMP9 expression increased in aortic root specimens in 8- and 52-week-old mice, and this increase was more apparent in 52-week-old mice. Additionally, the elevation of MMP9 and disordered collagen structure were evident in 52-week-old mice but not in high-fat-fed 8-week-old mice. Resveratrol prevented these changes in both groups of mice. This suggests that the inflammatory state was more pronounced in aged mice, suggesting that resveratrol might exert protective effects against aging-related vascular inflammation.

Platelets play an important role in thrombosis- and atherosclerosis-associated inflammation [30, 44]. In the present study, we found that MMP3 and MMP9 secretion by platelets participates in atherosclerosis. A previous study found that the expression of TLR4-positive circulating monocytes increased in patients with acute coronary syndrome and coronary atherosclerosis compared with the control group [45]. Silvello et al. reported that serum MMP9 levels were significantly higher in patients with atherosclerotic disease and peripheral vascular disease, but they did not find significant differences in serum MMP3 levels [46]. Beaudeux et al. also reported that serum MMP3 and MMP9 levels were significantly higher in subjects with hyperlipidemia and high cardiovascular risk [47]. In the present study, plasma MMP3 levels did not significantly increase in high-fat-fed mice, suggesting a likely biological difference between humans and mice.

The aging body often has chronic inflammation. This study observed that the expression of platelet senescence-related proteins p21 and p53 is increased when TLR4 signal is activated. However, the mechanism of inflammation affecting senescence is not yet clear. Resveratrol reduces inflammation while also reducing the expression of aging-related proteins p21 and p53, which also indicates that inflammation is closely related to aging.

The limitation of the present study is that it did not identify the role of TLR2. Since TLR2 and TLR4 have something in common. Both are associated with MyD88 and NF-*κ*B pathways in LPS-induced inflammatory response. However, the signals of TLR2 and TLR4 are also different. Pam3CSK4 is a specific TLR2 agonist but not TLR4. Tunjungputri et al. reported that LPS and Pam3CSK4 have different effects on IL-6 and IL-1*β* in activated platelets [48]. In the present study, we found that the action of resveratrol is multitargeted on inhibition of platelet function. Whether the action of resveratrol on inhibition of secretion of IL-1*β* in ox-LDL-stimulated platelets is related to TLR2 activation remains to be further explored.

In conclusion, the present study found that resveratrol inhibited the expression of TLR4 and secretion of MMP3, MMP9, and IL-1*β*. The mechanism of action of resveratrol appeared to be associated with the inhibition of TLR4/Syk/NLRP3 activation in ox-LDL-activated platelets. Furthermore, TLR4, MMP3, and MMP9 expression was higher in 52-week-old mice than in 8-week-old mice. Disordered vascular structure and vascular cell senescence were more apparent in 52-week-old mice compared with 8-week-old mice. Resveratrol treatment prevented these changes in vascular structure and vascular cell senescence and decreased TLR4, MMP3, and MMP9 expression in both groups of mice. These findings suggest that resveratrol-induced inhibition of the inflammatory response occurs through several molecular targets that involve platelet activation. Overall, resveratrol may be able to protect against aging-related vascular inflammation.

## Data Availability

The data underlying the findings of the paper are publicly available.

## Supplementary Materials

Supplementary Materials Supplemental Figure S1: resveratrol inhibited Syk phosphorylation in LPS-treated platelets. (A) Resveratrol dose-dependently inhibited Syk phosphorylation that was induced by LPS. (B) The Syk inhibitor R788 inhibited MMP3 and MMP9 expression that was induced by LPS. (C) R788 (30 nM) and resveratrol (10 *μ*M) synergistically inhibited MMP3 and MMP9 expression that was induced by LPS. The data represent three independent experiments. ^#^*p* < 0.05, ^##^*p* < 0.01, significant difference between non-LPS-treated platelets and LPS-treated platelets; ^∗^*p* < 0.05, ^∗∗^*p* < 0.01, significant difference between R788-treated platelets and LPS-activated platelets. Supplemental Figure S2: resveratrol suppressed NLRP3/caspase-1/IL-1*β* expression in LPS-treated platelets. (A) Resveratrol inhibited NLRP3, caspase-1, and IL-1*β* expression that was induced by LPS. (B) The NLRP3 inhibitor MCC950 inhibited caspase-1 and IL-1*β* expression that was induced by LPS. (C) MCC950 (100 nM) inhibited IL-1*β* secretion that was induced by LPS. (D) MCC950 inhibited caspase-1, IL-1*β*, MMP3, and MMP9 expression that was induced by LPS. (E) The combination of resveratrol (10 *μ*M) and MCC950 (10 nM) synergistically inhibited the expression of caspase-1, IL-1*β*, MMP3, and MMP9 in LPS-activated platelets. The data represent three independent experiments. ^#^*p* < 0.05, ^##^*p* < 0.01, significant difference between non-LPS-treated platelets and LPS-treated platelets; ^∗^*p* < 0.05, ^∗∗^*p* < 0.01, significant difference between MCC950- or MCC950+resveratrol-treated platelets and LPS-activated platelets.
