# Supplementary material for: Resveratrol Confers Vascular Protection by Suppressing TLR4/Syk/NLRP3 Signaling in Oxidized Low-Density Lipoprotein-Activated Platelets
Source: Oxid Med Cell Longev. 2021 Feb 25;2021:8819231. doi: 10.1155/2021/8819231 (PMC7935581; doi:10.1155/2021/8819231)
Supplement: Supplementary Materials — Supplemental Figure S1: resveratrol inhibited Syk phosphorylation in LPS-treated platelets. (A) Resveratrol dose-dependently inhibited Syk phosphorylation that was induced by LPS. (B) The Syk inhibitor R788 inhibited MMP3 and MMP9 expression that was induced by LPS. (C) R788 (30 nM) and resveratrol (10 μM) synergistically inhibited MMP3 and MMP9 expression that was induced by LPS. The data represent three independent experiments. #p < 0.05, ##p < 0.01, significant difference between non-LPS-treated platelets and LPS-treated platelets; ∗p < 0.05, ∗∗p < 0.01, significant difference between R788-treated platelets and LPS-activated platelets. Supplemental Figure S2: resveratrol suppressed NLRP3/caspase-1/IL-1β expression in LPS-treated platelets. (A) Resveratrol inhibited NLRP3, caspase-1, and IL-1β expression that was induced by LPS. (B) The NLRP3 inhibitor MCC950 inhibited caspase-1 and IL-1β expression that was induced by LPS. (C) MCC950 (100 nM) inhibited IL-1β secretion that was induced by LPS. (D) MCC950 inhibited caspase-1, IL-1β, MMP3, and MMP9 expression that was induced by LPS. (E) The combination of resveratrol (10 μM) and MCC950 (10 nM) synergistically inhibited the expression of caspase-1, IL-1β, MMP3, and MMP9 in LPS-activated platelets. The data represent three independent experiments. #p < 0.05, ##p < 0.01, significant difference between non-LPS-treated platelets and LPS-treated platelets; ∗p < 0.05, ∗∗p < 0.01, significant difference between MCC950- or MCC950+resveratrol-treated platelets and LPS-activated platelets. [file 8819231.f1.zip › Supplemental figure ledends.docx]

**Supplemental figure legends**

**Supplemental Fig. S1. Resveratrol inhibited Syk phosphorylation in LPS-treated platelets.** (A) Resveratrol dose-dependently inhibited Syk phosphorylation that was induced by LPS. (B) The Syk inhibitor R788 inhibited MMP3 and MMP9 expression that was induced by LPS. (C) R788 (30 nM) and resveratrol (10 μM) synergistically inhibited MMP3 and MMP9 expression that was induced by LPS. The data represent three independent experiments. ^#^*p* < 0.05, ^##^*p* < 0.01, significant difference between non-LPS-treated platelets and LPS-treated platelets; **p* < 0.05, ***p* < 0.01, significant difference between R788-treated platelets and LPS-activated platelets.

**Supplemental Fig. S2. Resveratrol suppressed NLRP3/caspase-1/IL-1β expression in LPS-treated platelets.** (A) Resveratrol inhibited NLRP3, caspase-1, and IL-1β expression that was induced by LPS. (B) The NLRP3 inhibitor MCC950 inhibited caspase-1 and IL-1β expression that was induced by LPS. (C) MCC950 (100 nM) inhibited IL-1β secretion that was induced by LPS. (D) MCC950 inhibited caspase-1, IL-1β, MMP3, and MMP9 expression that was induced by LPS. (E) The combination of resveratrol (10 μM) and MCC950 (10 nM) synergistically inhibited the expression of caspase-1, IL-1β, MMP3, and MMP9 in LPS-activated platelets. The data represent three independent experiments. ^#^*p* < 0.05, ^##^*p* < 0.01, significant difference between non-LPS-treated platelets and LPS-treated platelets; **p* < 0.05, ***p* < 0.01, significant difference between MCC950- or MCC950+resveratrol-treated platelets and LPS-activated platelets.
